# Supplementary material for: Hot spot prediction in protein-protein interactions by an ensemble system
Source: BMC Syst Biol. 2018 Dec 31;12(Suppl 9):132. doi: 10.1186/s12918-018-0665-8 (PMC6311905; doi:10.1186/s12918-018-0665-8)
Supplement: Supplementary file 2 — All relevant MATLAB code. A simple Matlab implement of our predictor. (ZIP 19,742 kb) [file 12918_2018_665_MOESM2_ESM.zip › Additional file 2/Read ASA/ASA website/dbMPIKT/4JEUA.html]

 NetSurfP - Protein Surface Accessibility and Secondary Structure Predictions

  

|  |  |  |
| --- | --- | --- |
|  |  | NetSurfP - Protein Surface Accessibility and Secondary Structure Predictions  Technical University of Denmark |

  


---

```
# For publication of results, please cite:
# A generic method for assignment of reliability scores applied to solvent accessibility predictions.
# Bent Petersen, Thomas Nordahl Petersen, Pernille Andersen, Morten Nielsen and Claus Lundegaard
# BMC Structural Biology 2009, 9:51 doi:10.1186/1472-6807-9-51
#
# Column 1: Class assignment - B for buried or E for Exposed - Threshold: 25% exposure, but not based on RSA
# Column 2: Amino acid
# Column 3: Sequence name
# Column 4: Amino acid number
# Column 5: Relative Surface Accessibility - RSA
# Column 6: Absolute Surface Accessibility
# Column 7: Z-fit score for RSA prediction
# Column 8: Probability for Alpha-Helix
# Column 9: Probability for Beta-strand
# Column 10: Probability for Coil
E P  4JEUA                  1    0.756 107.305  -1.295   0.000   0.001   0.999
E I  4JEUA                  2    0.484  89.596  -2.101   0.058   0.017   0.925
E G  4JEUA                  3    0.490  38.555   0.095   0.321   0.003   0.675
B L  4JEUA                  4    0.150  27.538  -1.306   0.923   0.002   0.076
E K  4JEUA                  5    0.278  57.143   1.013   0.970   0.001   0.030
E A  4JEUA                  6    0.555  61.183   1.037   0.970   0.001   0.030
B V  4JEUA                  7    0.199  30.602   1.705   0.970   0.001   0.030
B V  4JEUA                  8    0.032   4.888   0.609   0.970   0.001   0.030
B G  4JEUA                  9    0.249  19.604   1.283   0.970   0.001   0.030
E E  4JEUA                 10    0.526  91.910   1.444   0.970   0.001   0.030
E K  4JEUA                 11    0.306  63.006   1.931   0.970   0.001   0.030
B I  4JEUA                 12    0.025   4.643   0.470   0.970   0.001   0.030
B M  4JEUA                 13    0.138  27.694   1.121   0.988   0.000   0.012
E H  4JEUA                 14    0.503  91.550   1.588   0.970   0.001   0.030
E D  4JEUA                 15    0.320  46.069   1.750   0.970   0.001   0.030
B V  4JEUA                 16    0.018   2.828   1.131   0.970   0.001   0.030
B I  4JEUA                 17    0.105  19.444  -0.174   0.923   0.002   0.076
E K  4JEUA                 18    0.580 119.306   1.578   0.923   0.002   0.076
B K  4JEUA                 19    0.129  26.576  -0.043   0.858   0.002   0.139
B V  4JEUA                 20    0.045   6.978   0.189   0.600   0.003   0.397
E K  4JEUA                 21    0.626 128.830   0.957   0.406   0.004   0.590
E K  4JEUA                 22    0.692 142.447   0.916   0.176   0.004   0.820
E K  4JEUA                 23    0.322  66.277  -0.954   0.053   0.005   0.942
E G  4JEUA                 24    0.646  50.848  -1.245   0.018   0.019   0.964
E E  4JEUA                 25    0.434  75.872   0.209   0.005   0.045   0.951
B W  4JEUA                 26    0.127  30.592   1.112   0.004   0.420   0.576
B K  4JEUA                 27    0.094  19.254   0.513   0.001   0.900   0.099
B V  4JEUA                 28    0.032   4.857   0.525   0.001   0.959   0.040
B L  4JEUA                 29    0.015   2.820   1.851   0.001   0.959   0.040
B V  4JEUA                 30    0.026   3.919   0.733   0.001   0.959   0.040
B V  4JEUA                 31    0.025   3.781   1.273   0.001   0.900   0.099
B D  4JEUA                 32    0.052   7.536   0.574   0.004   0.420   0.576
E Q  4JEUA                 33    0.628 112.161   1.368   0.321   0.003   0.675
E L  4JEUA                 34    0.438  80.198   0.795   0.502   0.002   0.495
B S  4JEUA                 35    0.045   5.286  -0.365   0.600   0.003   0.397
E M  4JEUA                 36    0.305  61.031   0.742   0.782   0.003   0.216
E R  4JEUA                 37    0.502 115.004   1.001   0.858   0.002   0.139
B M  4JEUA                 38    0.107  21.391   0.514   0.923   0.002   0.076
B L  4JEUA                 39    0.042   7.672  -0.791   0.970   0.001   0.030
E S  4JEUA                 40    0.288  33.718  -0.172   0.923   0.002   0.076
E S  4JEUA                 41    0.323  37.902   0.208   0.938   0.007   0.055
B C  4JEUA                 42    0.089  12.524  -0.278   0.879   0.010   0.111
B C  4JEUA                 43    0.089  12.510   0.790   0.622   0.015   0.363
E K  4JEUA                 44    0.455  93.511   1.038   0.115   0.016   0.868
B M  4JEUA                 45    0.209  41.901   0.664   0.717   0.014   0.269
E T  4JEUA                 46    0.468  64.953   0.052   0.879   0.010   0.111
E D  4JEUA                 47    0.264  38.028   0.887   0.923   0.002   0.076
B I  4JEUA                 48    0.050   9.158  -0.326   0.938   0.007   0.055
E M  4JEUA                 49    0.343  68.714   0.960   0.879   0.010   0.111
E T  4JEUA                 50    0.689  95.578   1.014   0.802   0.014   0.185
E E  4JEUA                 51    0.268  46.750   2.024   0.455   0.046   0.498
E G  4JEUA                 52    0.452  35.580  -0.129   0.113   0.087   0.800
B I  4JEUA                 53    0.031   5.772   0.582   0.021   0.279   0.699
E T  4JEUA                 54    0.291  40.403   0.606   0.021   0.756   0.223
B I  4JEUA                 55    0.165  30.451   0.936   0.018   0.846   0.136
B V  4JEUA                 56    0.138  21.287   0.605   0.018   0.846   0.136
B E  4JEUA                 57    0.145  25.366   0.522   0.018   0.846   0.136
E D  4JEUA                 58    0.381  54.830   0.585   0.023   0.655   0.322
B I  4JEUA                 59    0.079  14.615   0.174   0.022   0.359   0.619
E N  4JEUA                 60    0.575  84.239   0.114   0.056   0.142   0.802
E K  4JEUA                 61    0.564 116.077   0.315   0.052   0.084   0.864
E R  4JEUA                 62    0.674 154.346  -0.220   0.053   0.043   0.903
E R  4JEUA                 63    0.388  88.760  -0.207   0.018   0.047   0.935
E E  4JEUA                 64    0.629 109.921  -0.256   0.018   0.047   0.935
E P  4JEUA                 65    0.551  78.229  -0.285   0.018   0.019   0.964
B L  4JEUA                 66    0.228  41.802  -0.952   0.018   0.019   0.964
E P  4JEUA                 67    0.617  87.496  -0.187   0.018   0.019   0.964
E S  4JEUA                 68    0.541  63.452  -1.027   0.005   0.045   0.951
B L  4JEUA                 69    0.111  20.306   0.964   0.004   0.138   0.858
E E  4JEUA                 70    0.365  63.765   0.464   0.005   0.262   0.733
B A  4JEUA                 71    0.026   2.909   0.788   0.001   0.900   0.099
B V  4JEUA                 72    0.017   2.644   1.548   0.000   0.983   0.017
B Y  4JEUA                 73    0.029   6.240   0.953   0.000   0.983   0.017
B L  4JEUA                 74    0.021   3.845   1.063   0.000   0.983   0.017
B I  4JEUA                 75    0.020   3.645   1.071   0.001   0.900   0.099
B T  4JEUA                 76    0.206  28.572   0.944   0.007   0.526   0.467
B P  4JEUA                 77    0.162  22.917  -0.184   0.005   0.015   0.979
E S  4JEUA                 78    0.293  34.293   0.616   0.016   0.005   0.979
E E  4JEUA                 79    0.456  79.646  -0.138   0.923   0.002   0.076
E K  4JEUA                 80    0.648 133.314   1.069   0.923   0.002   0.076
B S  4JEUA                 81    0.072   8.427   0.158   0.923   0.002   0.076
B V  4JEUA                 82    0.026   3.919   0.902   0.970   0.001   0.030
E H  4JEUA                 83    0.495  89.968   1.832   0.970   0.001   0.030
E S  4JEUA                 84    0.290  34.000   2.449   0.970   0.001   0.030
B L  4JEUA                 85    0.017   3.113   1.350   0.970   0.001   0.030
B I  4JEUA                 86    0.104  19.185   0.619   0.970   0.001   0.030
E S  4JEUA                 87    0.561  65.691   1.597   0.970   0.001   0.030
B D  4JEUA                 88    0.226  32.595   0.537   0.970   0.001   0.030
B F  4JEUA                 89    0.043   8.570   0.083   0.858   0.002   0.139
E K  4JEUA                 90    0.500 102.768   0.797   0.782   0.003   0.216
E D  4JEUA                 91    0.578  83.290   0.118   0.502   0.002   0.495
E P  4JEUA                 92    0.451  63.954  -0.295   0.321   0.003   0.675
E P  4JEUA                 93    0.537  76.200  -1.880   0.246   0.004   0.750
E T  4JEUA                 94    0.425  58.878  -0.403   0.181   0.016   0.803
E A  4JEUA                 95    0.511  56.345  -0.691   0.113   0.043   0.844
E K  4JEUA                 96    0.410  84.316   0.164   0.052   0.084   0.864
B Y  4JEUA                 97    0.101  21.605  -0.094   0.020   0.205   0.775
E R  4JEUA                 98    0.356  81.593   0.509   0.004   0.420   0.576
B A  4JEUA                 99    0.168  18.503   0.386   0.002   0.816   0.182
B A  4JEUA                100    0.027   2.931   0.319   0.001   0.959   0.040
B H  4JEUA                101    0.042   7.603   0.809   0.001   0.959   0.040
B V  4JEUA                102    0.020   3.074   1.102   0.001   0.959   0.040
B F  4JEUA                103    0.043   8.590   0.137   0.001   0.959   0.040
B F  4JEUA                104    0.030   6.021  -0.033   0.001   0.900   0.099
B T  4JEUA                105    0.051   7.101   0.505   0.003   0.718   0.279
B D  4JEUA                106    0.144  20.750  -0.826   0.005   0.336   0.660
E S  4JEUA                107    0.344  40.364  -0.288   0.018   0.088   0.893
B C  4JEUA                108    0.128  18.027   0.467   0.005   0.015   0.979
E P  4JEUA                109    0.298  42.229  -0.285   0.016   0.005   0.979
E D  4JEUA                110    0.575  82.829   0.234   0.923   0.002   0.076
E A  4JEUA                111    0.471  51.948   0.342   0.923   0.002   0.076
B L  4JEUA                112    0.053   9.668   0.315   0.970   0.001   0.030
B F  4JEUA                113    0.129  25.971   0.361   0.970   0.001   0.030
E N  4JEUA                114    0.507  74.283   1.316   0.970   0.001   0.030
E E  4JEUA                115    0.288  50.349   1.893   0.970   0.001   0.030
B L  4JEUA                116    0.027   4.962   0.417   0.923   0.002   0.076
E V  4JEUA                117    0.357  54.886   0.661   0.923   0.002   0.076
E K  4JEUA                118    0.672 138.292   0.966   0.782   0.003   0.216
E S  4JEUA                119    0.339  39.719  -0.215   0.406   0.004   0.590
E R  4JEUA                120    0.600 137.492  -1.359   0.176   0.004   0.820
E A  4JEUA                121    0.423  46.604  -0.790   0.053   0.005   0.942
B A  4JEUA                122    0.236  26.040  -0.653   0.053   0.043   0.903
E K  4JEUA                123    0.521 107.170  -0.872   0.019   0.141   0.840
E V  4JEUA                124    0.333  51.197   0.662   0.022   0.359   0.619
B I  4JEUA                125    0.097  17.908  -0.355   0.021   0.756   0.223
E K  4JEUA                126    0.288  59.303   0.593   0.018   0.846   0.136
E T  4JEUA                127    0.377  52.359   1.735   0.011   0.918   0.071
B L  4JEUA                128    0.036   6.610   0.239   0.011   0.918   0.071
B T  4JEUA                129    0.198  27.449   0.960   0.006   0.962   0.032
B E  4JEUA                130    0.186  32.442  -1.002   0.001   0.900   0.099
B I  4JEUA                131    0.050   9.232   1.527   0.002   0.816   0.182
B N  4JEUA                132    0.162  23.746  -0.284   0.004   0.420   0.576
B I  4JEUA                133    0.040   7.400   0.037   0.004   0.197   0.799
B A  4JEUA                134    0.134  14.712  -1.022   0.004   0.420   0.576
B F  4JEUA                135    0.041   8.209   0.187   0.004   0.616   0.381
B L  4JEUA                136    0.074  13.604   0.341   0.023   0.655   0.322
B P  4JEUA                137    0.047   6.740   0.348   0.022   0.552   0.426
B Y  4JEUA                138    0.142  30.410   0.168   0.005   0.336   0.660
E E  4JEUA                139    0.284  49.597  -0.513   0.005   0.045   0.951
E S  4JEUA                140    0.418  49.013  -0.750   0.003   0.003   0.994
E Q  4JEUA                141    0.308  54.937  -0.985   0.005   0.015   0.979
B V  4JEUA                142    0.077  11.820  -0.564   0.021   0.451   0.528
B Y  4JEUA                143    0.021   4.445   0.863   0.021   0.756   0.223
B S  4JEUA                144    0.055   6.434  -0.706   0.018   0.846   0.136
B L  4JEUA                145    0.028   5.200   0.346   0.003   0.718   0.279
E D  4JEUA                146    0.242  34.930  -0.675   0.004   0.514   0.481
B S  4JEUA                147    0.050   5.837   0.034   0.004   0.085   0.910
E A  4JEUA                148    0.361  39.738  -0.557   0.018   0.019   0.964
E D  4JEUA                149    0.397  57.164  -1.796   0.115   0.016   0.868
B S  4JEUA                150    0.078   9.142  -1.626   0.321   0.003   0.675
B F  4JEUA                151    0.078  15.715  -0.855   0.622   0.015   0.363
E Q  4JEUA                152    0.359  64.082  -0.105   0.622   0.015   0.363
E S  4JEUA                153    0.286  33.484  -0.309   0.622   0.015   0.363
B F  4JEUA                154    0.056  11.319  -0.657   0.522   0.016   0.462
B Y  4JEUA                155    0.141  30.132  -0.694   0.339   0.016   0.645
B S  4JEUA                156    0.260  30.495  -1.204   0.181   0.016   0.803
E P  4JEUA                157    0.435  61.726  -1.264   0.181   0.016   0.803
E H  4JEUA                158    0.489  89.022  -1.572   0.181   0.016   0.803
E K  4JEUA                159    0.331  68.169  -1.114   0.181   0.016   0.803
E A  4JEUA                160    0.433  47.739  -1.372   0.246   0.004   0.750
E Q  4JEUA                161    0.610 108.857  -0.683   0.502   0.002   0.495
E M  4JEUA                162    0.312  62.411  -0.713   0.502   0.002   0.495
B K  4JEUA                163    0.159  32.706  -1.220   0.694   0.003   0.303
E N  4JEUA                164    0.399  58.414  -0.438   0.858   0.002   0.139
E P  4JEUA                165    0.481  68.183   0.559   0.923   0.002   0.076
B I  4JEUA                166    0.106  19.628   0.317   0.923   0.002   0.076
B L  4JEUA                167    0.032   5.841   0.019   0.970   0.001   0.030
E E  4JEUA                168    0.377  65.844   0.965   0.970   0.001   0.030
E R  4JEUA                169    0.302  69.250   1.398   0.970   0.001   0.030
B L  4JEUA                170    0.029   5.347   0.409   0.970   0.001   0.030
B A  4JEUA                171    0.049   5.422  -0.255   0.970   0.001   0.030
E E  4JEUA                172    0.414  72.273   1.086   0.970   0.001   0.030
B Q  4JEUA                173    0.061  10.966   0.354   0.970   0.001   0.030
B I  4JEUA                174    0.029   5.365  -0.054   0.970   0.001   0.030
B A  4JEUA                175    0.159  17.478   0.127   0.970   0.001   0.030
E T  4JEUA                176    0.266  36.853   0.389   0.970   0.001   0.030
B L  4JEUA                177    0.028   5.054   0.315   0.970   0.001   0.030
B C  4JEUA                178    0.064   8.915   0.369   0.970   0.001   0.030
E A  4JEUA                179    0.336  37.038   0.479   0.970   0.001   0.030
E T  4JEUA                180    0.281  38.919  -0.289   0.858   0.002   0.139
B L  4JEUA                181    0.127  23.327  -0.066   0.694   0.003   0.303
E K  4JEUA                182    0.524 107.828  -0.188   0.109   0.005   0.886
E E  4JEUA                183    0.379  66.194  -0.079   0.018   0.019   0.964
B Y  4JEUA                184    0.187  39.941   1.034   0.004   0.085   0.910
B P  4JEUA                185    0.156  22.108  -0.549   0.004   0.197   0.799
B A  4JEUA                186    0.217  23.924   0.233   0.002   0.816   0.182
B V  4JEUA                187    0.032   4.918  -0.627   0.001   0.959   0.040
B R  4JEUA                188    0.179  40.877   0.541   0.001   0.959   0.040
B Y  4JEUA                189    0.068  14.510  -1.264   0.001   0.900   0.099
E R  4JEUA                190    0.396  90.592   0.585   0.004   0.514   0.481
E G  4JEUA                191    0.359  28.285  -1.295   0.004   0.085   0.910
E E  4JEUA                192    0.596 104.174  -1.396   0.018   0.019   0.964
E Y  4JEUA                193    0.496 105.952  -1.805   0.109   0.005   0.886
E K  4JEUA                194    0.514 105.627  -0.537   0.694   0.003   0.303
B D  4JEUA                195    0.192  27.653  -0.219   0.782   0.003   0.216
B N  4JEUA                196    0.038   5.622  -2.066   0.923   0.002   0.076
E A  4JEUA                197    0.403  44.400   1.337   0.970   0.001   0.030
E L  4JEUA                198    0.325  59.489   1.612   0.970   0.001   0.030
B L  4JEUA                199    0.023   4.156   1.238   0.970   0.001   0.030
B A  4JEUA                200    0.040   4.364  -0.388   0.988   0.000   0.012
E Q  4JEUA                201    0.501  89.496   1.661   0.988   0.000   0.012
B L  4JEUA                202    0.211  38.689   1.699   0.970   0.001   0.030
B I  4JEUA                203    0.023   4.200   0.892   0.970   0.001   0.030
E Q  4JEUA                204    0.267  47.650   1.438   0.970   0.001   0.030
E D  4JEUA                205    0.560  80.754   1.831   0.970   0.001   0.030
B K  4JEUA                206    0.160  32.933   1.212   0.970   0.001   0.030
B L  4JEUA                207    0.056  10.180   0.150   0.970   0.001   0.030
E D  4JEUA                208    0.555  79.961   1.241   0.970   0.001   0.030
E A  4JEUA                209    0.584  64.401   1.357   0.970   0.001   0.030
B Y  4JEUA                210    0.165  35.282   0.094   0.923   0.002   0.076
E K  4JEUA                211    0.350  72.036  -0.296   0.858   0.002   0.139
E A  4JEUA                212    0.653  71.961   0.007   0.782   0.003   0.216
E D  4JEUA                213    0.493  71.070  -0.782   0.694   0.003   0.303
E D  4JEUA                214    0.330  47.596  -0.904   0.321   0.003   0.675
E P  4JEUA                215    0.618  87.680  -1.819   0.176   0.004   0.820
E T  4JEUA                216    0.490  68.018  -1.819   0.115   0.016   0.868
B M  4JEUA                217    0.204  40.840  -1.490   0.058   0.017   0.925
E G  4JEUA                218    0.447  35.218  -2.106   0.058   0.017   0.925
E E  4JEUA                219    0.545  95.177  -1.757   0.058   0.017   0.925
E G  4JEUA                220    0.443  34.833  -2.188   0.058   0.017   0.925
E P  4JEUA                221    0.557  78.996  -1.781   0.058   0.017   0.925
E D  4JEUA                222    0.600  86.446  -1.608   0.058   0.017   0.925
E K  4JEUA                223    0.499 102.562  -1.316   0.018   0.019   0.964
E A  4JEUA                224    0.556  61.227  -0.749   0.018   0.019   0.964
E R  4JEUA                225    0.257  58.967   0.832   0.004   0.085   0.910
B S  4JEUA                226    0.082   9.622  -1.397   0.005   0.336   0.660
B Q  4JEUA                227    0.089  15.878   0.668   0.002   0.816   0.182
B L  4JEUA                228    0.015   2.728   1.510   0.001   0.900   0.099
B L  4JEUA                229    0.015   2.655   1.806   0.001   0.959   0.040
B I  4JEUA                230    0.021   3.811   1.067   0.001   0.900   0.099
B L  4JEUA                231    0.026   4.724   0.674   0.018   0.846   0.136
B D  4JEUA                232    0.054   7.853  -0.931   0.022   0.552   0.426
B R  4JEUA                233    0.141  32.358   0.329   0.018   0.088   0.893
E G  4JEUA                234    0.342  26.915  -0.934   0.018   0.047   0.935
B F  4JEUA                235    0.134  26.793   0.128   0.018   0.047   0.935
B D  4JEUA                236    0.143  20.621  -0.930   0.053   0.043   0.903
B P  4JEUA                237    0.104  14.701  -0.105   0.052   0.084   0.864
B S  4JEUA                238    0.083   9.681  -0.830   0.118   0.150   0.732
B S  4JEUA                239    0.060   7.055   0.009   0.191   0.086   0.723
B P  4JEUA                240    0.038   5.350  -1.329   0.307   0.165   0.527
B V  4JEUA                241    0.039   5.994  -0.680   0.278   0.093   0.628
B L  4JEUA                242    0.074  13.604  -0.485   0.278   0.093   0.628
B H  4JEUA                243    0.067  12.169  -0.177   0.268   0.043   0.689
B E  4JEUA                244    0.142  24.772  -0.739   0.522   0.016   0.462
B L  4JEUA                245    0.124  22.723   0.006   0.522   0.016   0.462
B T  4JEUA                246    0.086  11.887   0.459   0.522   0.016   0.462
B F  4JEUA                247    0.043   8.690   0.319   0.858   0.002   0.139
B Q  4JEUA                248    0.125  22.396  -0.062   0.938   0.007   0.055
B A  4JEUA                249    0.054   6.006  -1.300   0.938   0.007   0.055
B M  4JEUA                250    0.031   6.283   0.588   0.938   0.007   0.055
B S  4JEUA                251    0.022   2.567   1.112   0.938   0.007   0.055
E Y  4JEUA                252    0.260  55.498   0.406   0.879   0.010   0.111
B D  4JEUA                253    0.191  27.581  -0.501   0.802   0.014   0.185
B L  4JEUA                254    0.070  12.762  -0.485   0.751   0.050   0.199
B L  4JEUA                255    0.108  19.830   0.358   0.538   0.173   0.289
E P  4JEUA                256    0.433  61.443   0.492   0.199   0.152   0.649
B I  4JEUA                257    0.099  18.389   0.057   0.113   0.087   0.800
E E  4JEUA                258    0.646 112.839   0.416   0.052   0.084   0.864
E N  4JEUA                259    0.496  72.570  -0.532   0.005   0.045   0.951
E D  4JEUA                260    0.383  55.133  -1.408   0.004   0.138   0.858
E V  4JEUA                261    0.388  59.574   0.723   0.002   0.816   0.182
B Y  4JEUA                262    0.073  15.600   0.487   0.001   0.959   0.040
E K  4JEUA                263    0.500 102.871   0.930   0.001   0.959   0.040
B Y  4JEUA                264    0.089  19.126   1.049   0.002   0.816   0.182
E E  4JEUA                265    0.494  86.319   0.358   0.004   0.616   0.381
E T  4JEUA                266    0.384  53.275  -0.641   0.005   0.262   0.733
E S  4JEUA                267    0.672  78.770  -1.137   0.018   0.088   0.893
E G  4JEUA                268    0.575  45.292  -1.844   0.018   0.088   0.893
E I  4JEUA                269    0.546 100.955  -1.652   0.018   0.047   0.935
E G  4JEUA                270    0.626  49.243  -2.114   0.018   0.047   0.935
E E  4JEUA                271    0.621 108.506  -0.270   0.053   0.043   0.903
E A  4JEUA                272    0.569  62.737  -0.622   0.019   0.141   0.840
E R  4JEUA                273    0.512 117.340  -0.280   0.022   0.359   0.619
E V  4JEUA                274    0.532  81.830   0.501   0.022   0.552   0.426
E K  4JEUA                275    0.402  82.691   0.748   0.021   0.756   0.223
E E  4JEUA                276    0.471  82.231   0.520   0.018   0.846   0.136
B V  4JEUA                277    0.225  34.552   0.591   0.018   0.846   0.136
E L  4JEUA                278    0.327  59.947  -0.489   0.004   0.616   0.381
B L  4JEUA                279    0.091  16.607  -0.241   0.005   0.262   0.733
E D  4JEUA                280    0.471  67.813  -0.441   0.018   0.047   0.935
E E  4JEUA                281    0.596 104.121  -1.343   0.058   0.017   0.925
E D  4JEUA                282    0.530  76.344  -0.250   0.109   0.005   0.886
B D  4JEUA                283    0.087  12.537  -0.993   0.176   0.004   0.820
E D  4JEUA                284    0.582  83.837   1.102   0.923   0.002   0.076
B L  4JEUA                285    0.225  41.124   0.446   0.923   0.002   0.076
B W  4JEUA                286    0.088  21.260   0.618   0.970   0.001   0.030
E I  4JEUA                287    0.297  54.908   0.755   0.975   0.003   0.022
E A  4JEUA                288    0.606  66.803   1.181   0.938   0.007   0.055
B L  4JEUA                289    0.123  22.521   0.534   0.858   0.002   0.139
B R  4JEUA                290    0.203  46.395   0.483   0.694   0.003   0.303
E H  4JEUA                291    0.542  98.590  -1.584   0.406   0.004   0.590
E K  4JEUA                292    0.353  72.571   0.922   0.321   0.003   0.675
E H  4JEUA                293    0.287  52.114  -0.035   0.321   0.003   0.675
B I  4JEUA                294    0.059  10.952   0.973   0.858   0.002   0.139
E A  4JEUA                295    0.406  44.686   0.812   0.923   0.002   0.076
E E  4JEUA                296    0.512  89.359   1.497   0.970   0.001   0.030
B V  4JEUA                297    0.029   4.442   0.082   0.970   0.001   0.030
B S  4JEUA                298    0.062   7.220   0.115   0.970   0.001   0.030
E Q  4JEUA                299    0.619 110.625   1.614   0.970   0.001   0.030
E E  4JEUA                300    0.360  62.927   2.107   0.970   0.001   0.030
B V  4JEUA                301    0.018   2.705   1.370   0.988   0.000   0.012
E T  4JEUA                302    0.318  44.120   1.789   0.988   0.000   0.012
E R  4JEUA                303    0.659 150.888   1.797   0.988   0.000   0.012
B S  4JEUA                304    0.205  24.003   1.413   0.988   0.000   0.012
B L  4JEUA                305    0.023   4.285   0.752   0.988   0.000   0.012
E K  4JEUA                306    0.555 114.081   1.952   0.988   0.000   0.012
E D  4JEUA                307    0.516  74.327   2.198   0.988   0.000   0.012
B F  4JEUA                308    0.042   8.510   0.713   0.970   0.001   0.030
E S  4JEUA                309    0.313  36.742   1.421   0.970   0.001   0.030
E S  4JEUA                310    0.698  81.770   1.504   0.970   0.001   0.030
E S  4JEUA                311    0.482  56.526   0.943   0.923   0.002   0.076
B K  4JEUA                312    0.160  33.015   0.721   0.858   0.002   0.139
E R  4JEUA                313    0.629 143.995   0.771   0.858   0.002   0.139
E M  4JEUA                314    0.568 113.697   0.158   0.782   0.003   0.216
E N  4JEUA                315    0.297  43.466  -1.034   0.694   0.003   0.303
E T  4JEUA                316    0.488  67.630  -0.325   0.502   0.002   0.495
E G  4JEUA                317    0.615  48.385  -0.082   0.321   0.003   0.675
E E  4JEUA                318    0.540  94.408  -0.512   0.406   0.004   0.590
E K  4JEUA                319    0.545 112.004  -0.687   0.600   0.003   0.397
E T  4JEUA                320    0.511  70.834   0.194   0.522   0.016   0.462
E T  4JEUA                321    0.281  39.016   0.332   0.339   0.016   0.645
B M  4JEUA                322    0.138  27.594   0.092   0.923   0.002   0.076
E R  4JEUA                323    0.607 138.980   1.196   0.970   0.001   0.030
E D  4JEUA                324    0.426  61.415   1.569   0.970   0.001   0.030
B L  4JEUA                325    0.028   5.182   0.548   0.970   0.001   0.030
B S  4JEUA                326    0.235  27.495   0.971   0.970   0.001   0.030
E Q  4JEUA                327    0.530  94.694   1.522   0.970   0.001   0.030
B M  4JEUA                328    0.095  19.070   0.519   0.970   0.001   0.030
B L  4JEUA                329    0.042   7.654  -0.883   0.970   0.001   0.030
E K  4JEUA                330    0.505 103.940   0.772   0.923   0.002   0.076
E K  4JEUA                331    0.416  85.612   0.668   0.858   0.002   0.139
B M  4JEUA                332    0.060  12.066   1.100   0.858   0.002   0.139
E P  4JEUA                333    0.470  66.736   0.527   0.858   0.002   0.139
E Q  4JEUA                334    0.573 102.391   0.895   0.858   0.002   0.139
B Y  4JEUA                335    0.114  24.298   0.318   0.923   0.002   0.076
E Q  4JEUA                336    0.404  72.226   0.837   0.970   0.001   0.030
E K  4JEUA                337    0.624 128.357   0.871   0.970   0.001   0.030
E E  4JEUA                338    0.354  61.826   0.094   0.970   0.001   0.030
E L  4JEUA                339    0.330  60.350   0.469   0.923   0.002   0.076
E S  4JEUA                340    0.606  71.058   0.658   0.858   0.002   0.139
E K  4JEUA                341    0.298  61.340   1.416   0.923   0.002   0.076
B Y  4JEUA                342    0.055  11.796   0.523   0.970   0.001   0.030
E S  4JEUA                343    0.420  49.271   0.900   0.970   0.001   0.030
E T  4JEUA                344    0.286  39.668   1.443   0.970   0.001   0.030
B H  4JEUA                345    0.048   8.731   0.507   0.988   0.000   0.012
B L  4JEUA                346    0.097  17.724   0.410   0.970   0.001   0.030
E H  4JEUA                347    0.388  70.614   0.953   0.970   0.001   0.030
B L  4JEUA                348    0.044   7.965   0.225   0.988   0.000   0.012
B A  4JEUA                349    0.027   2.942  -0.040   0.970   0.001   0.030
E E  4JEUA                350    0.373  65.111   0.435   0.970   0.001   0.030
E D  4JEUA                351    0.353  50.838   1.081   0.970   0.001   0.030
B C  4JEUA                352    0.032   4.479  -0.269   0.970   0.001   0.030
B M  4JEUA                353    0.148  29.615   0.587   0.970   0.001   0.030
E K  4JEUA                354    0.573 117.887   1.499   0.988   0.000   0.012
E H  4JEUA                355    0.242  44.020   1.358   0.970   0.001   0.030
B Y  4JEUA                356    0.030   6.475   0.403   0.970   0.001   0.030
E Q  4JEUA                357    0.363  64.778   1.208   0.923   0.002   0.076
E G  4JEUA                358    0.618  48.644   1.354   0.858   0.002   0.139
E T  4JEUA                359    0.269  37.324   1.013   0.694   0.003   0.303
B V  4JEUA                360    0.043   6.609  -0.023   0.923   0.002   0.076
E D  4JEUA                361    0.406  58.562   0.441   0.970   0.001   0.030
E K  4JEUA                362    0.509 104.763   1.390   0.970   0.001   0.030
B L  4JEUA                363    0.073  13.293   0.041   0.988   0.000   0.012
B C  4JEUA                364    0.079  11.021  -0.044   0.970   0.001   0.030
E R  4JEUA                365    0.448 102.684   1.834   0.970   0.001   0.030
B V  4JEUA                366    0.057   8.792   0.794   0.970   0.001   0.030
B E  4JEUA                367    0.044   7.739  -0.050   0.970   0.001   0.030
B Q  4JEUA                368    0.170  30.326  -0.064   0.970   0.001   0.030
B D  4JEUA                369    0.256  36.818   0.037   0.970   0.001   0.030
B L  4JEUA                370    0.045   8.313   0.490   0.938   0.007   0.055
B A  4JEUA                371    0.077   8.474  -0.770   0.879   0.010   0.111
E M  4JEUA                372    0.283  56.728  -0.040   0.622   0.015   0.363
E G  4JEUA                373    0.495  38.988  -0.903   0.257   0.016   0.727
B T  4JEUA                374    0.287  39.835  -0.242   0.115   0.016   0.868
E D  4JEUA                375    0.398  57.395  -0.643   0.113   0.043   0.844
E A  4JEUA                376    0.490  54.053  -1.249   0.268   0.043   0.689
E E  4JEUA                377    0.569  99.369  -0.960   0.257   0.016   0.727
E G  4JEUA                378    0.375  29.489  -0.604   0.406   0.004   0.590
E E  4JEUA                379    0.598 104.436  -0.377   0.522   0.016   0.462
E K  4JEUA                380    0.516 106.162   0.206   0.430   0.016   0.555
B I  4JEUA                381    0.111  20.591  -0.137   0.622   0.015   0.363
E K  4JEUA                382    0.402  82.630   0.213   0.782   0.003   0.216
E D  4JEUA                383    0.550  79.226   0.478   0.858   0.002   0.139
B P  4JEUA                384    0.109  15.410   0.010   0.923   0.002   0.076
B M  4JEUA                385    0.039   7.864   0.177   0.970   0.001   0.030
E R  4JEUA                386    0.475 108.844   1.403   0.970   0.001   0.030
E A  4JEUA                387    0.383  42.174   1.711   0.970   0.001   0.030
B I  4JEUA                388    0.035   6.420   0.206   0.970   0.001   0.030
B V  4JEUA                389    0.146  22.440   0.916   0.970   0.001   0.030
E P  4JEUA                390    0.548  77.775   1.148   0.923   0.002   0.076
B I  4JEUA                391    0.140  25.900   0.396   0.923   0.002   0.076
B L  4JEUA                392    0.077  14.044  -0.826   0.782   0.003   0.216
E L  4JEUA                393    0.472  86.332  -0.555   0.600   0.003   0.397
E D  4JEUA                394    0.494  71.142  -0.503   0.246   0.004   0.750
E A  4JEUA                395    0.590  65.062  -0.621   0.053   0.005   0.942
E N  4JEUA                396    0.744 108.863  -0.041   0.003   0.003   0.994
B V  4JEUA                397    0.132  20.227   0.375   0.003   0.003   0.994
E S  4JEUA                398    0.487  57.112   0.745   0.016   0.005   0.979
B T  4JEUA                399    0.244  33.898   0.178   0.923   0.002   0.076
E Y  4JEUA                400    0.252  53.959   0.211   0.923   0.002   0.076
B D  4JEUA                401    0.135  19.454   0.713   0.970   0.001   0.030
B K  4JEUA                402    0.062  12.856   1.110   0.970   0.001   0.030
B I  4JEUA                403    0.018   3.312   1.465   0.970   0.001   0.030
B R  4JEUA                404    0.063  14.473   1.043   0.988   0.000   0.012
B I  4JEUA                405    0.023   4.329   1.023   0.988   0.000   0.012
B I  4JEUA                406    0.021   3.903   1.296   0.988   0.000   0.012
B L  4JEUA                407    0.014   2.600   1.515   0.988   0.000   0.012
B L  4JEUA                408    0.030   5.530   0.388   0.988   0.000   0.012
B Y  4JEUA                409    0.058  12.288   0.793   0.988   0.000   0.012
B I  4JEUA                410    0.032   6.012   0.100   0.970   0.001   0.030
B F  4JEUA                411    0.034   6.884  -0.079   0.923   0.002   0.076
B L  4JEUA                412    0.199  36.437   0.222   0.879   0.010   0.111
E K  4JEUA                413    0.412  84.790   0.203   0.406   0.004   0.590
E N  4JEUA                414    0.475  69.584  -0.257   0.109   0.005   0.886
E G  4JEUA                415    0.673  53.004  -0.954   0.016   0.005   0.979
B I  4JEUA                416    0.216  39.960  -0.342   0.016   0.005   0.979
E T  4JEUA                417    0.485  67.228   0.122   0.016   0.005   0.979
E E  4JEUA                418    0.594 103.824  -0.687   0.858   0.002   0.139
E E  4JEUA                419    0.673 117.503   0.778   0.923   0.002   0.076
B N  4JEUA                420    0.218  31.959   0.886   0.923   0.002   0.076
B L  4JEUA                421    0.063  11.590   0.053   0.970   0.001   0.030
E N  4JEUA                422    0.494  72.248   1.506   0.970   0.001   0.030
E K  4JEUA                423    0.453  93.141   1.851   0.988   0.000   0.012
B L  4JEUA                424    0.039   7.178   0.162   0.988   0.000   0.012
B I  4JEUA                425    0.190  35.113   0.817   0.970   0.001   0.030
E Q  4JEUA                426    0.649 115.911   0.800   0.923   0.002   0.076
E H  4JEUA                427    0.410  74.561   0.406   0.879   0.010   0.111
B A  4JEUA                428    0.197  21.665   0.416   0.406   0.004   0.590
E Q  4JEUA                429    0.742 132.503  -0.139   0.053   0.005   0.942
B I  4JEUA                430    0.098  18.185   0.844   0.003   0.003   0.994
E P  4JEUA                431    0.518  73.518   0.498   0.018   0.019   0.964
E P  4JEUA                432    0.533  75.590  -0.704   0.782   0.003   0.216
E E  4JEUA                433    0.508  88.748  -0.138   0.858   0.002   0.139
B D  4JEUA                434    0.082  11.759  -1.096   0.923   0.002   0.076
B S  4JEUA                435    0.207  24.237   0.273   0.970   0.001   0.030
E E  4JEUA                436    0.401  70.037   0.907   0.970   0.001   0.030
B I  4JEUA                437    0.057  10.600   0.961   0.970   0.001   0.030
B I  4JEUA                438    0.038   7.012  -0.086   0.970   0.001   0.030
E T  4JEUA                439    0.448  62.124   1.377   0.970   0.001   0.030
B N  4JEUA                440    0.244  35.795   0.248   0.970   0.001   0.030
B M  4JEUA                441    0.052  10.445   0.419   0.970   0.001   0.030
E A  4JEUA                442    0.368  40.598   0.664   0.923   0.002   0.076
E H  4JEUA                443    0.539  98.099   0.412   0.858   0.002   0.139
B L  4JEUA                444    0.141  25.835   0.241   0.694   0.003   0.303
E G  4JEUA                445    0.446  35.084  -1.707   0.321   0.003   0.675
E V  4JEUA                446    0.342  52.519  -1.208   0.176   0.004   0.820
E P  4JEUA                447    0.527  74.739  -1.074   0.113   0.043   0.844
E I  4JEUA                448    0.344  63.658  -1.359   0.113   0.087   0.800
B V  4JEUA                449    0.266  40.838  -0.561   0.052   0.084   0.864
E T  4JEUA                450    0.471  65.369  -0.536   0.113   0.087   0.800
E D  4JEUA                451    0.611  88.045  -1.732   0.113   0.043   0.844
E S  4JEUA                452    0.497  58.295  -1.903   0.113   0.043   0.844
E T  4JEUA                453    0.455  63.136  -1.497   0.113   0.043   0.844
E L  4JEUA                454    0.385  70.494  -1.871   0.113   0.043   0.844
E R  4JEUA                455    0.402  92.035  -1.545   0.184   0.043   0.773
E R  4JEUA                456    0.451 103.279  -1.189   0.191   0.086   0.723
E R  4JEUA                457    0.476 108.912  -1.163   0.191   0.086   0.723
E S  4JEUA                458    0.411  48.204  -1.067   0.191   0.086   0.723
E K  4JEUA                459    0.351  72.201  -0.685   0.191   0.086   0.723
E P  4JEUA                460    0.404  57.384  -1.452   0.118   0.150   0.732
E E  4JEUA                461    0.559  97.587  -1.558   0.118   0.150   0.732
E R  4JEUA                462    0.393  90.066  -0.898   0.125   0.227   0.648
E K  4JEUA                463    0.514 105.750  -1.089   0.118   0.150   0.732
E E  4JEUA                464    0.536  93.569  -1.102   0.118   0.150   0.732
E R  4JEUA                465    0.484 110.905  -0.518   0.056   0.142   0.802
B I  4JEUA                466    0.282  52.096  -1.799   0.052   0.084   0.864
E S  4JEUA                467    0.619  72.559  -0.760   0.052   0.084   0.864
E E  4JEUA                468    0.581 101.466  -1.039   0.053   0.043   0.903
E Q  4JEUA                469    0.458  81.799  -0.980   0.118   0.150   0.732
E T  4JEUA                470    0.476  66.091  -0.853   0.135   0.317   0.548
B Y  4JEUA                471    0.253  54.023  -0.020   0.148   0.418   0.435
E Q  4JEUA                472    0.333  59.420  -0.702   0.148   0.418   0.435
B L  4JEUA                473    0.167  30.504  -0.475   0.216   0.235   0.548
B S  4JEUA                474    0.212  24.858  -1.530   0.118   0.150   0.732
E R  4JEUA                475    0.354  81.158  -0.971   0.052   0.084   0.864
B W  4JEUA                476    0.199  47.860  -0.718   0.113   0.087   0.800
E T  4JEUA                477    0.303  41.998   0.067   0.268   0.043   0.689
E P  4JEUA                478    0.455  64.579  -1.530   0.802   0.014   0.185
E I  4JEUA                479    0.308  56.888  -0.138   0.879   0.010   0.111
B I  4JEUA                480    0.053   9.842  -1.058   0.970   0.001   0.030
B K  4JEUA                481    0.203  41.778   0.567   0.970   0.001   0.030
E D  4JEUA                482    0.439  63.202   0.614   0.970   0.001   0.030
B I  4JEUA                483    0.071  13.209   0.506   0.988   0.000   0.012
B M  4JEUA                484    0.024   4.722   0.611   0.988   0.000   0.012
E E  4JEUA                485    0.345  60.289   1.103   0.988   0.000   0.012
E D  4JEUA                486    0.370  53.389   0.530   0.970   0.001   0.030
B T  4JEUA                487    0.064   8.932  -0.180   0.970   0.001   0.030
B I  4JEUA                488    0.140  25.974  -0.193   0.858   0.002   0.139
E E  4JEUA                489    0.672 117.468   1.108   0.694   0.003   0.303
E D  4JEUA                490    0.562  80.927  -1.476   0.246   0.004   0.750
E K  4JEUA                491    0.410  84.358  -0.295   0.109   0.005   0.886
B L  4JEUA                492    0.253  46.398  -0.986   0.058   0.017   0.925
E D  4JEUA                493    0.475  68.448  -0.163   0.058   0.017   0.925
E T  4JEUA                494    0.524  72.707  -0.460   0.115   0.016   0.868
E K  4JEUA                495    0.673 138.518  -0.881   0.113   0.087   0.800
E H  4JEUA                496    0.492  89.458  -0.843   0.056   0.142   0.802
B Y  4JEUA                497    0.157  33.530  -0.130   0.064   0.216   0.721
E P  4JEUA                498    0.356  50.445  -1.030   0.056   0.142   0.802
E Y  4JEUA                499    0.337  71.910  -0.909   0.066   0.296   0.638
B I  4JEUA                500    0.128  23.754  -2.058   0.066   0.296   0.638
E S  4JEUA                501    0.466  54.592  -1.321   0.064   0.216   0.721
E T  4JEUA                502    0.485  67.283  -1.836   0.056   0.142   0.802
E R  4JEUA                503    0.385  88.188  -1.189   0.052   0.084   0.864
E S  4JEUA                504    0.441  51.673  -2.303   0.053   0.043   0.903
E S  4JEUA                505    0.435  50.959  -1.910   0.115   0.016   0.868
E A  4JEUA                506    0.367  40.410  -2.682   0.115   0.016   0.868
E S  4JEUA                507    0.466  54.603  -2.291   0.115   0.016   0.868
E F  4JEUA                508    0.499 100.069  -2.377   0.113   0.043   0.844
E S  4JEUA                509    0.494  57.920  -2.354   0.113   0.043   0.844
E T  4JEUA                510    0.389  53.996  -2.039   0.053   0.043   0.903
E T  4JEUA                511    0.463  64.287  -1.943   0.052   0.084   0.864
E A  4JEUA                512    0.492  54.240  -1.425   0.052   0.084   0.864
E V  4JEUA                513    0.346  53.119  -1.564   0.052   0.084   0.864
E S  4JEUA                514    0.522  61.132  -1.816   0.052   0.084   0.864
E A  4JEUA                515    0.363  39.959  -1.878   0.113   0.087   0.800
E R  4JEUA                516    0.465 106.462  -1.611   0.113   0.087   0.800
E Y  4JEUA                517    0.344  73.513  -1.038   0.113   0.087   0.800
E G  4JEUA                518    0.375  29.512  -1.704   0.113   0.043   0.844
E H  4JEUA                519    0.477  86.839  -1.591   0.184   0.043   0.773
E W  4JEUA                520    0.399  95.935  -1.478   0.191   0.086   0.723
E H  4JEUA                521    0.399  72.669  -1.360   0.184   0.043   0.773
E K  4JEUA                522    0.507 104.331  -0.504   0.184   0.043   0.773
E N  4JEUA                523    0.643  94.121  -1.462   0.184   0.043   0.773
E K  4JEUA                524    0.487 100.258  -0.764   0.184   0.043   0.773
E A  4JEUA                525    0.403  44.455  -1.245   0.184   0.043   0.773
E P  4JEUA                526    0.494  70.084  -1.111   0.184   0.043   0.773
E G  4JEUA                527    0.537  42.262  -1.737   0.113   0.043   0.844
E E  4JEUA                528    0.577 100.749  -0.934   0.113   0.043   0.844
E Y  4JEUA                529    0.508 108.517  -1.908   0.113   0.043   0.844
E R  4JEUA                530    0.617 141.224  -1.169   0.058   0.017   0.925
E S  4JEUA                531    0.616  72.242  -0.274   0.018   0.019   0.964
E G  4JEUA                532    0.313  24.657  -0.463   0.018   0.019   0.964
E P  4JEUA                533    0.433  61.457   0.459   0.005   0.045   0.951
B R  4JEUA                534    0.194  44.403   0.899   0.004   0.616   0.381
B L  4JEUA                535    0.019   3.442   1.331   0.001   0.959   0.040
B I  4JEUA                536    0.017   3.071   1.752   0.000   0.983   0.017
B I  4JEUA                537    0.015   2.775   1.763   0.000   0.983   0.017
B F  4JEUA                538    0.022   4.415   1.438   0.000   0.983   0.017
B I  4JEUA                539    0.029   5.328   0.342   0.001   0.959   0.040
B L  4JEUA                540    0.044   8.056   0.016   0.001   0.900   0.099
B G  4JEUA                541    0.084   6.627  -1.100   0.005   0.336   0.660
B G  4JEUA                542    0.131  10.286  -2.140   0.004   0.085   0.910
B V  4JEUA                543    0.055   8.469  -0.773   0.018   0.047   0.935
B S  4JEUA                544    0.069   8.099  -0.126   0.018   0.019   0.964
B L  4JEUA                545    0.118  21.661   0.403   0.970   0.001   0.030
B N  4JEUA                546    0.158  23.175  -0.156   0.970   0.001   0.030
B E  4JEUA                547    0.029   5.136  -0.285   0.970   0.001   0.030
B M  4JEUA                548    0.033   6.623   0.029   0.970   0.001   0.030
B R  4JEUA                549    0.213  48.731   0.643   0.970   0.001   0.030
B C  4JEUA                550    0.070   9.772   0.191   0.970   0.001   0.030
B A  4JEUA                551    0.014   1.554   1.633   0.970   0.001   0.030
B Y  4JEUA                552    0.239  50.967   1.094   0.970   0.001   0.030
E E  4JEUA                553    0.348  60.883   1.372   0.970   0.001   0.030
B V  4JEUA                554    0.055   8.469   0.860   0.970   0.001   0.030
B T  4JEUA                555    0.099  13.731  -0.454   0.923   0.002   0.076
E Q  4JEUA                556    0.653 116.644   1.034   0.858   0.002   0.139
E A  4JEUA                557    0.575  63.376  -0.080   0.694   0.003   0.303
E N  4JEUA                558    0.425  62.176  -0.287   0.321   0.003   0.675
E G  4JEUA                559    0.653  51.375  -1.133   0.016   0.005   0.979
E K  4JEUA                560    0.590 121.363   0.265   0.005   0.015   0.979
B W  4JEUA                561    0.166  39.875   0.874   0.004   0.197   0.799
E E  4JEUA                562    0.332  58.000   1.354   0.003   0.718   0.279
B V  4JEUA                563    0.019   2.905   1.439   0.001   0.959   0.040
B L  4JEUA                564    0.021   3.827   1.207   0.000   0.983   0.017
B I  4JEUA                565    0.022   3.996   1.237   0.000   0.983   0.017
B G  4JEUA                566    0.020   1.558   0.481   0.001   0.900   0.099
B S  4JEUA                567    0.027   3.153  -0.383   0.022   0.552   0.426
B T  4JEUA                568    0.089  12.303  -0.376   0.022   0.359   0.619
B H  4JEUA                569    0.205  37.271   0.281   0.022   0.359   0.619
B I  4JEUA                570    0.045   8.288  -0.038   0.004   0.420   0.576
B L  4JEUA                571    0.088  16.149   0.149   0.005   0.262   0.733
B T  4JEUA                572    0.248  34.356   0.728   0.005   0.045   0.951
B P  4JEUA                573    0.063   8.926  -0.843   0.694   0.003   0.303
E Q  4JEUA                574    0.550  98.141   1.056   0.858   0.002   0.139
E K  4JEUA                575    0.447  91.886   1.807   0.923   0.002   0.076
B L  4JEUA                576    0.056  10.199   0.662   0.970   0.001   0.030
B L  4JEUA                577    0.050   9.118   0.643   0.970   0.001   0.030
E D  4JEUA                578    0.552  79.486   1.798   0.970   0.001   0.030
E T  4JEUA                579    0.283  39.210   0.984   0.970   0.001   0.030
B L  4JEUA                580    0.034   6.244   0.073   0.970   0.001   0.030
E K  4JEUA                581    0.428  87.957   0.970   0.923   0.002   0.076
E K  4JEUA                582    0.686 141.069   1.718   0.782   0.003   0.216
B L  4JEUA                583    0.097  17.797   0.288   0.406   0.004   0.590
E N  4JEUA                584    0.611  89.392   0.491   0.109   0.005   0.886
E K  4JEUA                585    0.486  99.950   1.495   0.109   0.005   0.886
E T  4JEUA                586    0.645  89.475   0.765   0.176   0.004   0.820
E D  4JEUA                587    0.441  63.606   0.251   0.257   0.016   0.727
E E  4JEUA                588    0.655 114.463   0.782   0.339   0.016   0.645
E E  4JEUA                589    0.587 102.514   1.073   0.257   0.016   0.727
E I  4JEUA                590    0.482  89.133  -0.952   0.003   0.003   0.994

Explain the output.  Go back.
```

---
